# Formation flight and collision avoidance for multiple UAVs based on modified tentacle algorithm in unstructured environments

**DOI:** 10.1371/journal.pone.0182006

**Published:** 2017-08-01

**Authors:** Minghuan Zhang

**Affiliations:** School of Astronautics, Northwestern Polytechnical University, Xi'an, Shaan xi Province, People’s Republic of China; Beihang University, CHINA

## Abstract

This paper presents a method for formation flight and collision avoidance of multiple UAVs. Due to the shortcomings such as collision avoidance caused by UAV’s high-speed and unstructured environments, this paper proposes a modified tentacle algorithm to ensure the high performance of collision avoidance. Different from the conventional tentacle algorithm which uses inverse derivation, the modified tentacle algorithm rapidly matches the radius of each tentacle and the steering command, ensuring that the data calculation problem in the conventional tentacle algorithm is solved. Meanwhile, both the speed sets and tentacles in one speed set are reduced and reconstructed so as to be applied to multiple UAVs. Instead of path iterative optimization, the paper selects the best tentacle to obtain the UAV collision avoidance path quickly. The simulation results show that the method presented in the paper effectively enhances the performance of flight formation and collision avoidance for multiple high-speed UAVs in unstructured environments.

## 1. Introduction

To control the formation flight of multiple unmanned air vehicles (UAVs) is a challenge as they are widely used for military and civil purposes [1–2]. Through the cooperation among multiple UAVs, their formation flight performances in missions such as search and rescue, surveillance, mapping and deployment of troops [3]are more effectively enhanced. The flight formation technique is the building block of multiple UAVs’ cooperation.

Collision avoidance is central to the UAV formation flight research [4–6]. Regarded as a complicated control problem, it faces challenges in designing a quick and robust controller which can maintain the relative position as well as safe distance in between [7]. In order to avoid collision between UAVs and obstacles or UAV pairs, it is urgently necessary to study formation switching and collision avoidance. Classical path planning methods, such as potential field method, genetic algorithm, grid-based method and geometric approach [8–19], are applied to single UAV collision avoidance. Many researchers have contributed to the development of collision avoidance algorithms for a single entity. Ref. [20] presents a modified artificial potential field (MAPF) method for a UAV to avoid collision in a 3D space. Due to the shortcomings of the traditional artificial potential field (APF) method, the MAPF method is developed in a certain constraint reference frame to decouple the decomposed force from the MAPF method with specific physical constraints. In the constraint reference frame, the path is examined with the updated force of the MAPF method, implemented by the UAV, and corrected if the updated force disagrees with the physical constraints. Such an examination and correction loop makes sure that the planned path can practically meet the UAV’s motion status and manoeuver capability. In order to enhance the estimation accuracy affected by the constantly changing path-loss factor during UAV flight, Ref. [21] proposed a UAV collision detection and decision making and path re-planning method.

In order to overcome the shortcomings of existing methods, this paper considers the manoeuver information of both UAV and aerial intruder and then presents a collision decision-making method based on the proposed regions and the interfered fluid dynamical (IFD) algorithm. Ref. [22] proposes a three-dimensional (3D) and real-time path planning method by combining the improved Lyapunov guidance vector field (LGVF) and the interfered fluid dynamical system (IFDS) with the varying receding-horizon optimization strategy based on the model predictive control (MPC).The experimental results show that the above hybrid method is applicable to various dynamic environments. Ref. [23] presents a collision avoidance algorithm for cooperative UAVs that share three-dimensional airspace. Based on the geometric optimization model, the feasible and optimal trajectory is obtained for a chosen UAV, with the local optimization scope reaching the operational level. The local optimization scope generates an optimal flight trajectory with the objective function in response to a set of restrictions that reduces the solution space. This collision avoidance manoeuver has such advantages as optimization with minimal cost, robustness that considers the global traffic condition, scalability that possesses explicit coordinates of waypoints and efficiency in implementing various tests of tuning parameters.

For multiple UAVs, it is necessary to consider their formation flight and collision avoidance simultaneously. Without taking into consideration the correlation between multiple UAVs, the collision avoidance method often works in a static structured environment and was not directly applied to the collision avoidance of formation flight. Ref. [24] proposes a guidance law for formation flight and collision avoidance. With the concept of elastic weighting factor, multiple UAVs are able to actively cope with the collision between both UAVs and static obstacles during their formation flight. Based on the sophisticated route planning, which spends time on processing environmental information, this guidance law has good collision avoidance performance. In a static structured environment, the UAV formation does not need to update environmental information frequently, and the UAVs have sufficient residual manoeuver time. However, when they fly at high speed in an unstructured environment, it is necessary to compute the information on environment and path in each time set so that the residual manoeuver time can be greatly shortened. Failure avoidance is highly possible when UAVs do not have enough time to complete their manoeuver. Its computation should be done in real time. The speed of UAV always needs to be over 100m/s to fulfil a specific mission, while the maximum speed in Ref. [24] is only 60m/s, even much less according to quadrotor in other articles. Therefore, these conventional methods have difficulties in computing in real time the collision avoidance of multiple high-speed UAVs in unstructured environments.

Felix proposed a simple but effective method for autonomous robot navigation in unstructured environments by using a set of “tentacles” that represents pre-calculated trajectories defined in the ego-centred occupancy grid [25]. To compute the collision avoidance in real time in unstructured environments, this method has the advantage of selection instead of real time computation. All the potential paths are pre-computed and stored; thus the real-time path planning becomes unnecessary. It is not necessary to create a whole environment model. This means that the cost of real-time computation will be greatly reduced. Therefore, we propose the method for collision avoidance of multiple high-speed UAVs under unstructured environments.

However, the speed of UAV is far greater than a robot in Ref. [25] and the steering command generation suffers from the data calculation problem the application problem. This paper proposes a method for multiple UAVs’ formation flight and collision avoidance based on the modified tentacle algorithm. Since the speed sets and tentacles in one speed set are reduced and reconstructed, the data calculation problem of the UAV are also solved. By modifying the ego-centred occupancy grid, we model their formation on an unstructured environment and solve the application problem. Instead of path computing, we can select the best tentacle to obtain the UAV collision avoidance path quickly. The simulation results show that our method can effectively compute in real time.

This paper is organized as follows. Section 2 proposes the formation flight controller. Section 3 gives the collision avoidance method based on the modified tentacle algorithm. Section 4 provides simulation results in which the performance of the proposed method is verified. Finally, Section 5 is devoted to the summary of the main results and the future work.

## 2. UAV formation flight

In order to show the dynamic and static obstacle avoidance simultaneously, this paper describe an UAV formation flight. For each single UAV, all the other UAVs are considered as a dynamic obstacle when the formation is avoiding a static obstacle.

**Fig 1:**
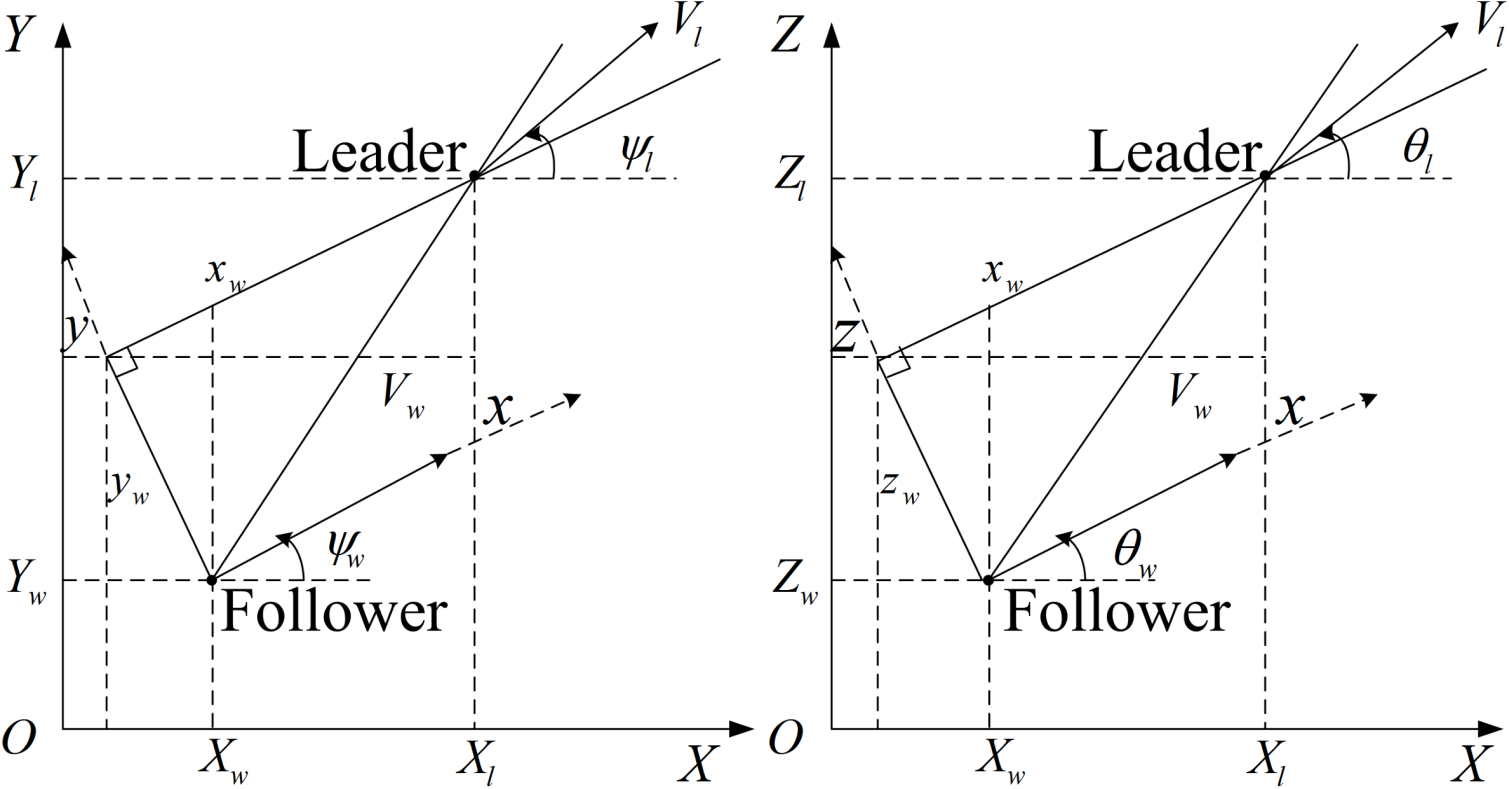
Inertial coordinate system and reference frame.

Each UAV in its formation is considered as a mass point. We use the leader-follower formation model[26–30]: one of the UAVs in the formation is defined as the leader and the other is the follower. Therefore, the formation control problem is transformed into a tracking problem between follower and leader. This means that the follower only needs to keep an appropriate relative position and direction from the leader. This paper takes follower as origin to establish reference frame on follower[31–34] to show the relationship between leader and follower, as Fig 1 shows.

*OXYZ* is the inertial coordinate system, *X*_*l*_,*Y*_*l*_,*Z*_*l*_ is the coordinate of the leader in inertial system, while *X*_*w*_,*Y*_*w*_,*Z*_*w*_ is the follower’s coordinate. In follower reference frame, *x*_*w*_,*y*_*w*_,*z*_*w*_ refers the distance between leader and follower. *V*_*l*_,*V*_*w*_ refers velocity of leader and follower. *ψ*_*l*_,*ψ*_*w*_ is the heading angle of leader and follower and *θ*_*l*_,*θ*_*w*_ is their track angle.

The motion of UAV is controlled by autopilot, its mathematical model[35–37] as Eq (1):

$$\begin{array}{l} {\dot{V}}_{l}=\frac{1}{\tau_{V_{l}}}(V_{lc}-V_{l})\\ {\dot{V}}_{w}=\frac{1}{\tau_{V_{w}}}(V_{wc}-V_{w})\\ {\dot{\psi }}_{l}=\frac{1}{\tau_{\psi_{l}}}(\psi_{lc}-\psi_{l})\\ {\dot{\psi }}_{w}=\frac{1}{\tau_{\psi_{w}}}(\psi_{wc}-\psi_{w})\\ {\dot{\theta }}_{l}=\frac{1}{\tau_{\theta_{l}}}(\theta_{lc}-\theta_{l})\\ {\dot{\theta }}_{w}=\frac{1}{\tau_{\theta_{w}}}(\theta_{wc}-\theta_{w}) \end{array}$$ (1)

In above equation, $\tau_{V_{l}},\tau_{V_{w}},\tau_{\psi_{l}},\tau_{\psi_{w}},\tau_{\theta_{l}},\tau_{\theta_{w}}$ refers to the time constant of velocity, heading angle, track angle, while *V*_*lc*_,*V*_*wc*_,*ψ*_*lc*_,*ψ*_*wc*_,*θ*_*lc*_,*θ*_*wc*_ refers to the instruction of velocity, heading angle, track angle.

Motion equation of leader and follower in inertial coordinate:

$$\begin{array}{l} {\dot{X}}_{l}=V_{l}\cos\psi_{l}\cos\theta_{l}\\ {\dot{Y}}_{l}=V_{l}\sin\psi_{l}\cos\theta_{l}\\ {\dot{Z}}_{l}=V_{l}\sin\theta_{l}\\ {\dot{X}}_{w}=V_{w}\cos\psi_{w}\cos\theta_{w}\\ {\dot{Y}}_{w}=V_{w}\sin\psi_{w}\cos\theta_{w}\\ {\dot{Z}}_{w}=V_{w}\sin\theta_{w} \end{array}$$ (2)

According to geometrical relationship in Fig 1, the coordinate in inertial frame of leader can display as follow:

$$\begin{array}{l} X_{l}=X_{w}+x_{w}\cos\psi_{w}\cos\theta_{w}-y_{w}\sin\psi_{w}+z_{w}\cos\psi_{w}\sin\theta_{w}\\ Y_{l}=Y_{w}+x_{w}\sin\psi_{w}\cos\theta_{w}+y_{w}\cos\psi_{w}+z_{w}\sin\psi_{w}\sin\theta_{w}\\ Z_{l}=Z_{w}+x_{w}\sin\theta_{w}+z_{w}\cos\theta_{w} \end{array}$$ (3)

Namely,

$$\left[\begin{array}{l} X_{l}-X_{w}\\ Y_{l}-Y_{w}\\ Z_{l}-Z_{w} \end{array}\right]=A\left[\begin{array}{l} x_{w}\\ y_{w}\\ z_{w} \end{array}\right]$$ (4)

Inside,

$$A=\left[\begin{array}{lll} \cos\psi_{w}\cos\theta_{w} & -\sin\psi_{w} & \cos\psi_{w}\sin\theta_{w}\\ \sin\psi_{w}\cos\theta_{w} & \cos\psi_{w} & \sin\psi_{w}\sin\theta_{w}\\ \sin\theta_{w} & 0 & \cos\theta_{w} \end{array}\right]$$ (5)

The relative distance between two UAVs in three dimensions as:

$$\left[\begin{array}{l} x_{w}\\ y_{w}\\ z_{w} \end{array}\right]=A^{-1}\left[\begin{array}{l} X_{l}-X_{w}\\ Y_{l}-Y_{w}\\ Z_{l}-Z_{w} \end{array}\right]$$ (6)

Then a PID formation controller can be designed to keep the distance in Eq (6) by computing a group of control instruction *V*_*wc*_,*ψ*_*wc*_,*θ*_*wc*_ for the follower.

The error between follower’s current position and expected position is as follows:

$$e=\left[\begin{array}{l} e_{x}\\ e_{y}\\ e_{z} \end{array}\right]=\left[\begin{array}{l} X_{l}-{\bar{X}}_{w}\\ Y_{l}-{\bar{Y}}_{w}\\ Z_{l}-{\bar{Z}}_{w} \end{array}\right]-\left[\begin{array}{l} X_{l}-X_{w}\\ Y_{l}-Y_{w}\\ Z_{l}-Z_{w} \end{array}\right]=A\left[\begin{array}{l} x_{w}\\ y_{w}\\ z_{w} \end{array}\right]-\left[\begin{array}{l} X_{l}-X_{w}\\ Y_{l}-Y_{w}\\ Z_{l}-Z_{w} \end{array}\right]$$ (7)

In above, ${\bar{X}}_{w},{\bar{Y}}_{w},{\bar{Z}}_{w}$ respect to follower’s expected position. Follower’s control instruction *V*_*wc*_,*ψ*_*wc*_,*θ*_*wc*_ can be generated by the PID algorithm:

$$\begin{array}{l} V_{wc}=K_{px}e_{x}+K_{ix}\int e_{x}dt+K_{dx}\frac{de_{x}}{dt}\\ \psi_{wc}=K_{py}e_{y}+K_{iy}\int e_{y}dt+K_{dy}\frac{de_{y}}{dt}\\ \theta_{wc}=K_{pz}e_{z}+K_{iz}\int e_{z}dt+K_{dz}\frac{de_{z}}{dt} \end{array}$$ (8)

## 3. Modified tentacle algorithm for collision avoidance

### 3.1. Basic tentacle algorithm

The primary purpose of this algorithm is to let the robot move within an unknown environment in a way similar to how a beetle crawls around and uses its antennae to avoid obstacles. Indeed, the basic idea consists of using a set of virtual antennae called tentacles that probe an ego-centred occupancy grid.

According to Ref.[25], the tentacle algorithm for an intelligent vehicle can be implemented as follows:

#### 3.1.1 Occupancy grid

**Fig 2:**
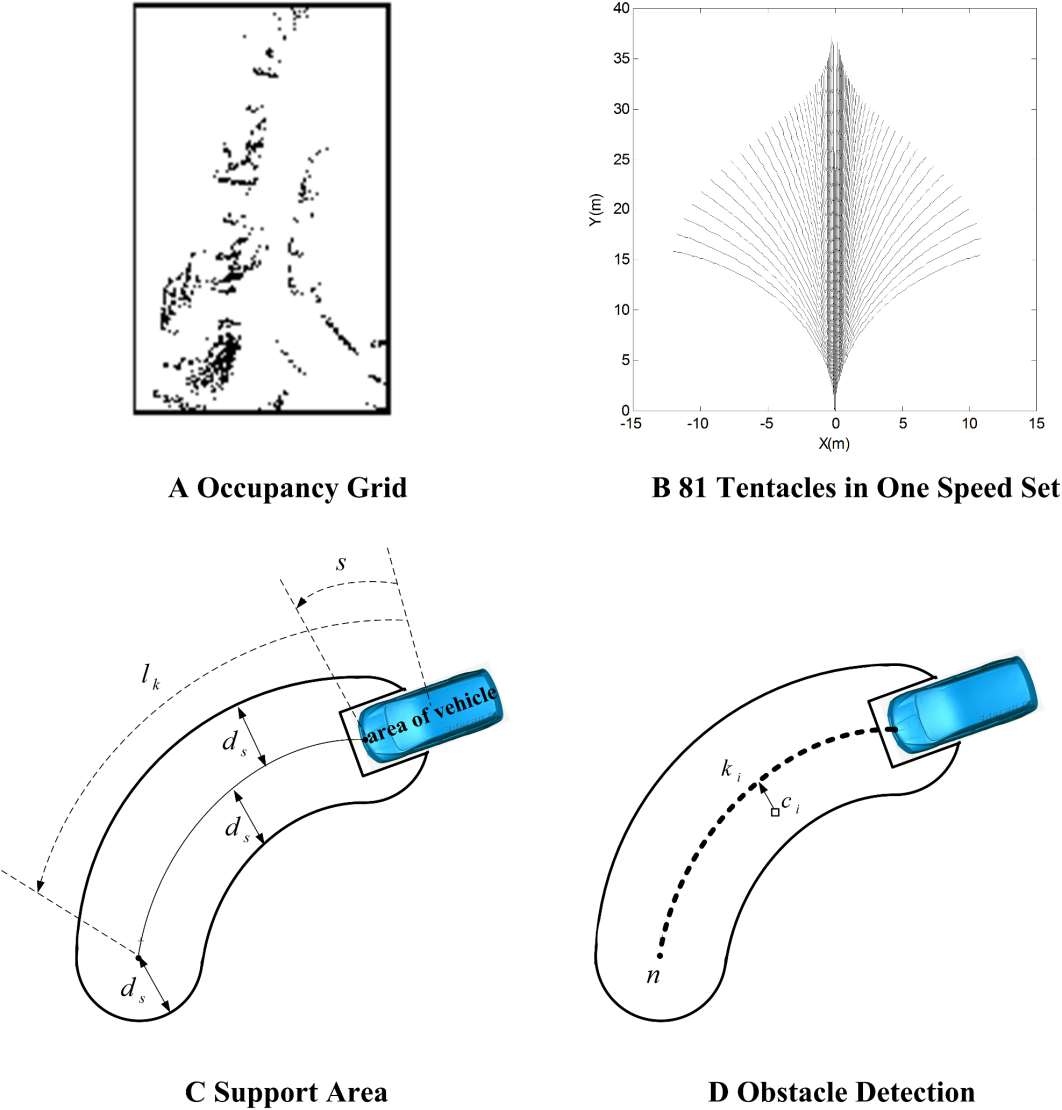
Basic tentacle algorithm.

Based on the information from a sensor, the environment around an intelligent vehicle can be described through a binary image as Fig 2A.The whole environment is divided into 512×512 pixel points, each of which is a cell covering a small ground patch of 25*cm*×25*cm*, the black points showing obstacles.

#### 3.1.2 Tentacle structure

Each tentacle is a potential path and 16 sets of tentacles are used. Each set contains 81 tentacles corresponding to a specific velocity of the vehicle, which is divided into 16 speed sets form 0 to10m/s. All tentacles are represented in the local coordinate system of the vehicle, start at the vehicle’s center of gravity and use the shape of circular arcs. The shape of 16×81 tentacles are designed to ensure an occupancy grid can be divided averagely.

The radius *r*_*k*_ of the *k* − *th* tentacle in a set is given by:

$$r_{k}=\left\{ \begin{array}{ll} \rho^{k}R_{j} & k=0,\ldots ,39\\ \infty & k=40\\ -\rho^{k-41}R_{j} & k=41,\ldots ,80 \end{array}\right.$$ (9)

where *ρ* is an exponential factor and *R*_*j*_ is the initial radius of speed, we set *j* = 0,…15.

The length of the *k* − *th* tentacle in a set is given by:

$$\begin{array}{l} l_{k}=\left\{ \begin{array}{ll} l+20\sqrt{\frac{k}{40}} & k=0,\ldots ,40\\ l+20\sqrt{\frac{k-40}{40}} & k=41,\ldots ,80 \end{array}\right.\\ l=8+33.5q^{1.2}\\ q=j/15 \end{array}$$ (10)

The velocity for speed set *j* is given by:

$$v_{j}=v_{s}+q^{1.2}(v_{e}-v_{s})$$ (11)

where *v*_*s*_ is the minimum speed and *v*_*e*_ is the maximum speed. Fig 2B shows the 81 tentacles in one speed set.

#### 3.1.3 Obstacle detection

The support area of a tentacle in the occupancy grid is used to determine whether a tentacle is drivable; its geometric definition is illustrated in Fig 2C. The **Rule A** of obstacle detection is as follows:

- Divide a tentacle to *n* sections as Fig 2D, and each point *c*_*i*_ in support area corresponds to a position *k*_*i*_;
- Count all the black points which show the obstacles and record their positions;
- Use an array *v*[*n*] to count the number of the black points in each section *k*_*i*_;
- Use a sliding window to determine the position of the first obstacle. The window is initially placed at *k*_0_ and successively slid to *k*_*n*−1_.If the sum of binary values within this window exceeds a threshold *n*_*o*_, an obstacle is detected and the position of the sliding window yields the distance *l*_*o*_ to the first obstacle.

#### 3.1.4 Tentacle selection and execution

Set the crash distance *l*_*c*_, which depends on the speed *v*, a deceleration *a* and a security distance *l*_*s*_:

$$l_{c}=l_{s}+\frac{v^{2}}{2a}$$ (12)

In the end, one best tentacle is selected as the expected trajectory with the three functions: *v*_*clearance*_,*v*_*flatness*_,*v*_*trajectory*_. The *v*_*clearance*_ depends on the distance to the first obstacle *l*_*o*_, the *v*_*flatness*_ has the goal to prefer tentacles leading over smooth terrain, and the *v*_*trajectory*_ pushes the vehicle towards following a given trajectory. The three function can be combined with different weight:

$$v_{combined}=a_{1}v_{clearance}+a_{2}v_{flatness}+a_{3}v_{trajectory}$$ (13)

where *a*_1_,*a*_2_,*a*_3_ are the weighting coefficients. The **Rule B** of tentacle selection is as follows:

- If there is no tentacle meet the condition *l*_*o*_ > *l*_*c*_, the vehicle must decelerate along the tentacle with the maximum *v*_*clearance*_ until the condition *l*_*o*_ > *l*_*c*_ is meet again;
- If there are many tentacles meet the condition *l*_*o*_ > *l*_*c*_, choose the tentacle with the minimum *v*_*combined*_.

**Fig 3:**
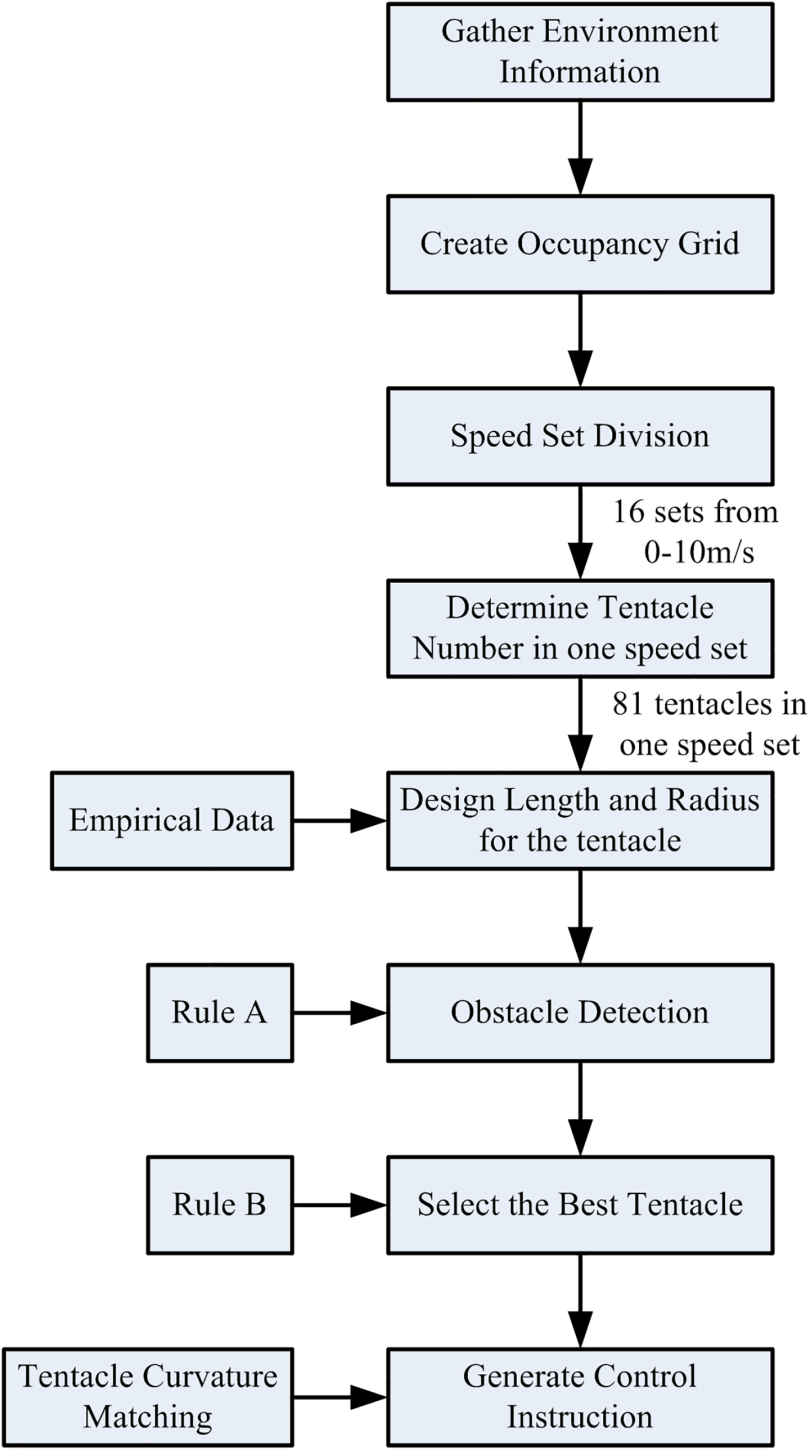
Basic tentacle algorithm.

The flow chart of the basic tentacle algorithm is as Fig 3.

#### 3.1.5 Problem in tentacle algorithm

**Problem 1:** It is noticed that all the parameters are related to wheel force and steering angle. This means that once a group of wheel forces and steering angles is certain, the trajectory of the vehicle may be fixed. Therefore, a group of wheel forces and steering angles refer to each tentacle. So the vehicle can be controlled to follow the selected tentacle with the two parameters.

**Fig 4:**
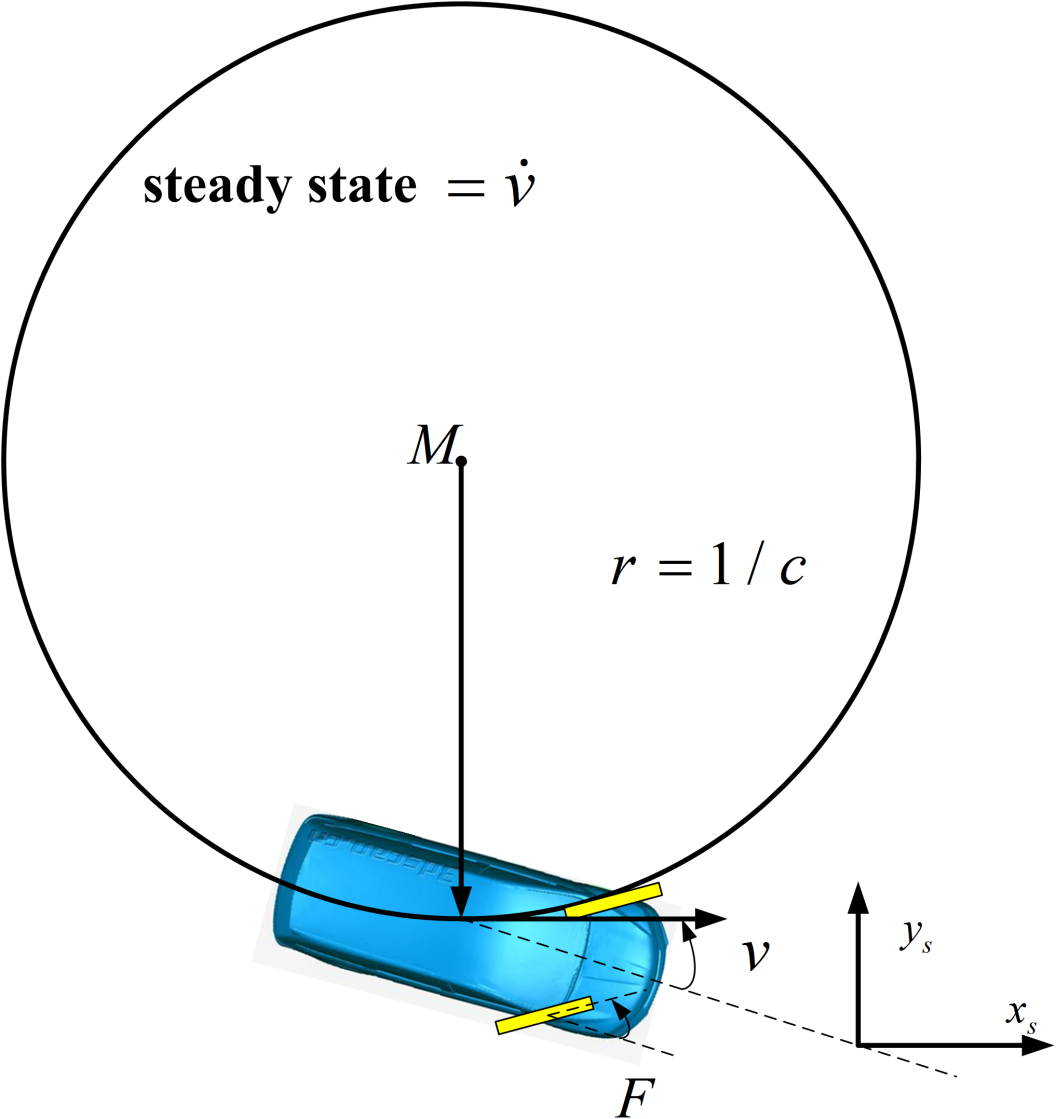
Steady state in tentacle algorithm.

Ref. [25] presents a method for executing the selected tentacle. This method pre-computes all the corresponding groups of wheel forces and steering angles through creating a steady state (as shown in Fig 4) according to each tentacle by curvature matching, and then stores them so that once a tentacle is selected, the corresponding wheel force and steering angle can be acquired at the same time.

Although this method is correct in theory, some problems may cause the tentacle algorithm invalid because when the radius of a tentacle is too large, the matching between this tentacle and the corresponding steady state costs plenty of time and still worst a steady state cannot be achieved.

**Problem 2:** The basic tentacle algorithm in Ref. [25] is applied to an autonomous robot although this algorithm needs to be modified on a UAV platform. The velocity of the UAV is much greater than the autonomous robot. So the radius of each tentacle should be greater to match the velocity. Meanwhile, this change results that most tentacles gather in a small area, so that many neighbouring tentacles will provide the same curvature. Thus, the number of tentacles in one speed set should be reduced so as to enhance the ability of real time computation for UAVs.

### 3.2. Modified tentacle algorithm

Although the UAV formation is in 3D, its collision avoidance can be considered as two 2D problems. Similar as Fig 1, we can project all the UAVs and obstacles to *XOY* and *XOZ* in inertial coordinate system, and design our modified tentacle algorithm in the two planes separately. We use the *XOY* plane here to show how the modify tentacle algorithm solves the two problems above.

The tentacle algorithm for UAV is modified as follows:

#### 3.2.1 Occupancy grid

**Fig 5:**
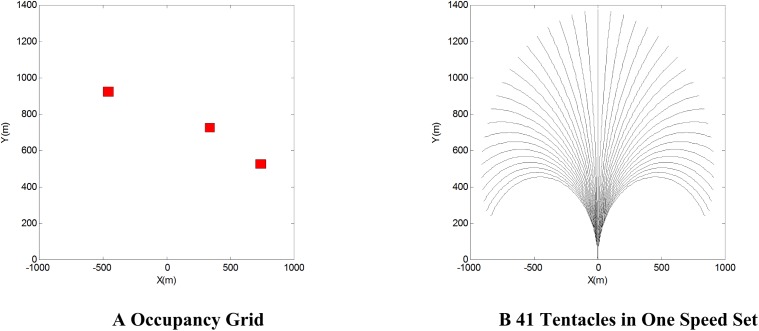
Modified occupancy grid and tentacles.

Since the area of flight airspace is much greater than the ground, the whole environment is divided into 1000×1400 pixel points, each pixel point is a cell which covers a ground patch of 1*m*×1*m*, the red area refers to the obstacles, as Fig 5A shows:

#### 3.2.2 Tentacle structure

In the basic tentacle algorithm, once the radius of each tentacle is fixed, the corresponding control instruction only can be obtained by matching a steady state with the same curvature, but it is hard to match when the tentacle has large radius. For solving this problem, different from determining the radius firstly for each tentacle, we consider using an inverse derivation method, firstly determine the manoeuver capacity for an UAV.

According to the manoeuver capacity of an UAV, the velocity of UAV from 50*m*/*s* to 150*m*/*s* is divided into 10 sets, and in one speed set, the horizontal overload capacity −2*g* to 2*g* is divided into 41 sets. Therefore there are 10×41 tentacles totally, we consider this division ensure an occupancy grid can be divided averagely.

According to Newton’s second law, the radius *r* of a tentacle meets the following condition:

$$n_{y}=\frac{v_{y}^{2}}{r}$$ (14)

where *n*_*y*_ is the horizontal overload, *v*_*y*_ is the flight velocity in *XOY* plane. So the radius of each tentacle *r*_*k*_ in one speed set *j* can be computed by:

$$\begin{array}{l} r_{k}=\frac{v_{j}^{2}}{n_{yk}},k=0,\ldots ,40\\ n_{yk}=n_{y\min}+k\frac{n_{y\max}-n_{y\min}}{40}\\ v_{j}=v_{\min}+j\frac{v_{\max}-v_{\min}}{10},j=0,\ldots ,9 \end{array}$$ (15)

It should be noted that, both of the control instruction and the radius of tentacle are transferred to the functions with tentacle number *k*. It means once a tentacle is selected, instead of curvature matching in basic algorithm, the corresponding control instruction can be computed directly through reading the variable *k*. Thus, the modification can avoid the curvature matching in basic tentacle algorithm, and the Problem 1 can be solved in theory.

Similarly, the length *l*_*k*_ can be given by:

$$l_{k}=\left\{ \begin{array}{ll} 400\frac{j}{9}+200\sqrt{\frac{k}{20}} & k=0,\ldots ,20\\ 400\frac{j}{9}+200\sqrt{\frac{40-k}{20}} & k=21,\ldots ,40 \end{array}\right.$$ (16)

Fig 5B shows the 41 tentacles in one speed set. It can be seen that each tentacle divide the occupancy grid averagely. It proves the Problem 2 is solved in theory.

#### 3.2.3 Obstacle detection

The obstacle detection is similar to the basic tentacle algorithm, which, therefore, can be used for the UAV. The obstacle can be detected through **Rule A**.

It should be noted that, for an UAV in its formation, all the other UAVs are considered to be the dynamic obstacles in the occupancy grid, so this detection method may eliminate impact on the manoeuver of multiple UAVs.

#### 3.2.4 Tentacle selection

The crash distance *l*_*c*_ also is given by Eq (12), and the three function *v*_*clearance*_,*v*_*flatness*_,*v*_*trajectory*_ in Eq (13) are given by the following equations respectively.

*v*_*clearance*_ follows a normal distribution:

$$\begin{array}{l} v_{clearance}(l_{o})=\left\{ \begin{array}{l} 0\;if\;there\;is\;no\;obstacle\\ e^{-c_{1}l_{o}^{2}}\;otherwise \end{array}\right.\\ c_{1}=-\frac{\ln\frac{1}{2}}{l_{0.5}^{2}}\\ l_{0.5}=300 \end{array}$$ (17)

where *v*_*clearance*_ ∈ [0,1] has been normalization and meets the following conditions:

$$\begin{array}{l} v_{clearance}(0)=1\\ v_{clearance}(l_{0.5})=0.5\\ v_{clearance}(\infty )=0 \end{array}$$ (18)

This means that the smaller *v*_*clearance*_ shows the corresponding tentacle with a large distance from the next obstacle, so that the tentacles with small *v*_*clearance*_ are preferable.

Because the UAV motion is projected to *XOY* plane, the effect of *v*_*flatness*_ should be ignored:

$$v_{flatness}=0$$ (19)

Similar to *v*_*clearance*_, *v*_*trajectory*_ also follow a normal distribution:

$$\begin{array}{l} v_{trajectory}(k)=e^{-c_{3}{\left(k-k_{0}\right)}^{2}}-1\\ c_{3}=-\frac{\ln\;2}{1600} \end{array}$$ (20)

where *v*_*trajectory*_ ∈ [0,1] has been normalization and meet the following conditions:

$$\begin{array}{l} v_{trajectory}(k_{0}+40)=1\\ v_{trajectory}(0)=0 \end{array}$$ (21)

*k*_0_ can be obtained through matching the current overload instruction.

Using the previous three new functions, the evaluation index *v*_*combined*_ is calculated, then the best tentacle can be selected through **Rule B**, and we can compute the control instruction [*V*_*yc*_,*ψ*_*c*_] by Eq (15) with the number of the best tentacle.

Similarly in the *XOZ* plane, another control instruction [*V*_*zc*_,*θ*_*c*_] can be obtained. So the 3D control instruction for an UAV is [*V*_*c*_,*ψ*_*c*_,*θ*_*c*_], where $V_{c}=\sqrt{V_{yc}^{2}+V_{zc}^{2}}$.

**Fig 6:**
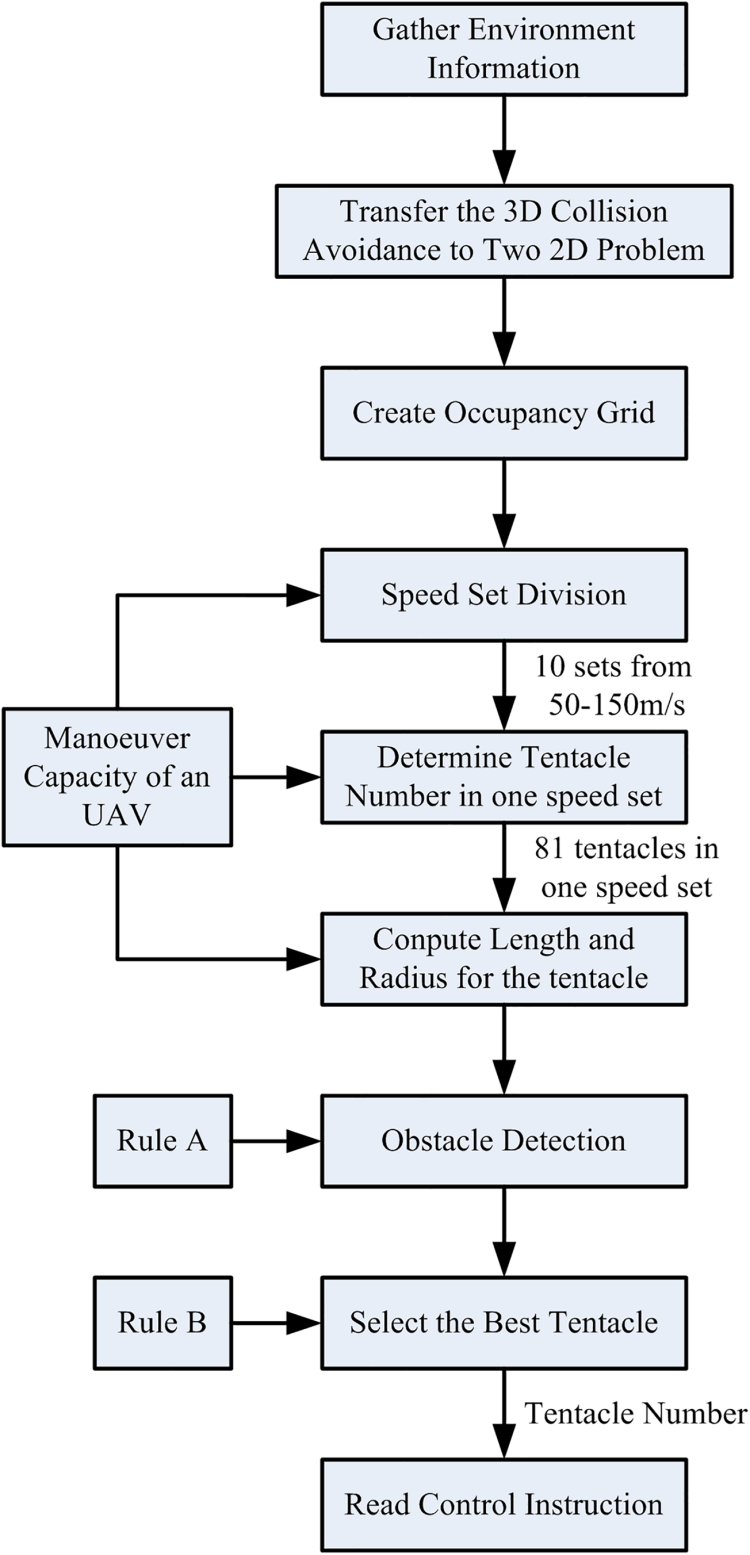
Modified tentacle algorithm.

The flow chart of the modified tentacle algorithm is as Fig 6.

#### 3.2.5 Problem solution in modified tentacle algorithm

**Problem 1:** Different from computing the steering command according to the radius of each tentacle with the basic tentacle algorithm, by using the inverse derivation, the modified tentacle algorithm divides the control instruction into some uniform sections, and then computes the radius of each tentacle with Newton's second law. It ensures each tentacle corresponds with only one overload instruction, so the curvature matching in basic algorithm become unnecessary. Thus, the data calculation problem(Problem 1) can be solved.

**Problem 2:** Because the speed sets and tentacles in one speed set are reduced and reconstructed, the influence of computation load in real time computation become insignificant. It ensures the modified tentacle algorithm meet the requirement of the real time computation for UAVs(Problem 2).

## 4. Experimental verification

### 4.1. Simulation environment

In order to verify the performance of the modified algorithm, a complex scenario with 3 obstacles and 5 UAVs is created in this paper.

The leader-follower formation model contains one leader and four followers. The initial states of each UAV are given in Table 1, where *τ*_*v*_,*τ*_*ψ*_,*τ*_*θ*_ represent the time constants of the autopilot.

**Table 1:** UAV’s initial states.

Parameters	Position(m)	Speed(m/s)	Heading angle(Degree)	Track angle(Degree)	*τ*_*v*_	*τ*_*ψ*_	*τ*_*θ*_
Leader	(500,200,190)	100	0	0	5	1	1
Follower#1	(0,0,100)	100	0	0	5	1	1
Follower#2	(0,300,300)	100	0	0	5	1	1
Follower#3	(0,-200,50)	100	0	0	5	1	1
Follower#4	(0,700,350)	100	0	0	5	1	1

Table 2 shows the relative distance in three dimensions between each follower and leader:

**Table 2:** Formation flight requirements.

Parameters	Relative Distance in the X Direction (m)	Relative Distance in the Y Direction (m)	Relative Distance in the Z Direction (m)
Leader	0	0	0
Follower#1	400	150	100
Follower#2	400	-150	-100
Follower#3	400	300	120
Follower#4	400	-300	-130

Table 3 gives the information on obstacles during the simulation, where *d*_*s*_ represents the requirements for safe distances in each tentacle.

**Table 3:** Obstacle parameters.

Parameters	Position(m)	Radius(m)	Safe distance d_s_ in tentacle(m)
Obstacle#1	(2000,100,100)	80	30
Obstacle#2	(4000,200,250)	80	30
Obstacle#3	(2000,500,330)	80	30

The simulation will be terminated when the leader UAV reach 10000m in x direction. The simulation step is 0.001s.

### 4.2. Simulation results and analysis

**Fig 7:**
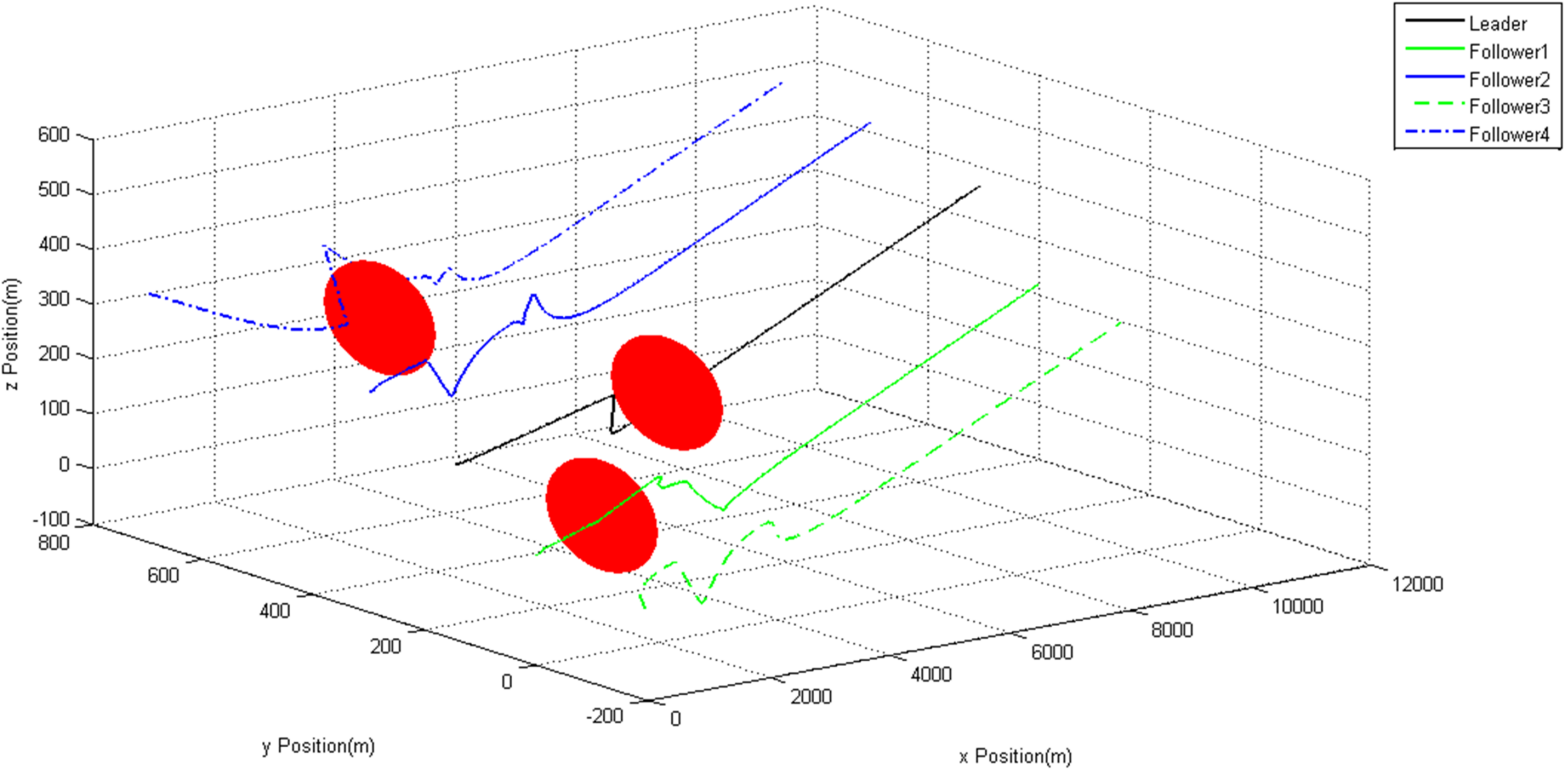
3D Trajectories of UAVs.

**Fig 8:**
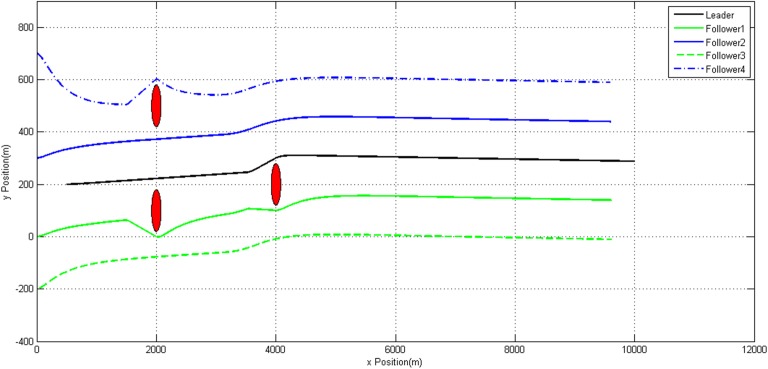
Trajectories of UAVs in *xoy* plane.

**Fig 9:**
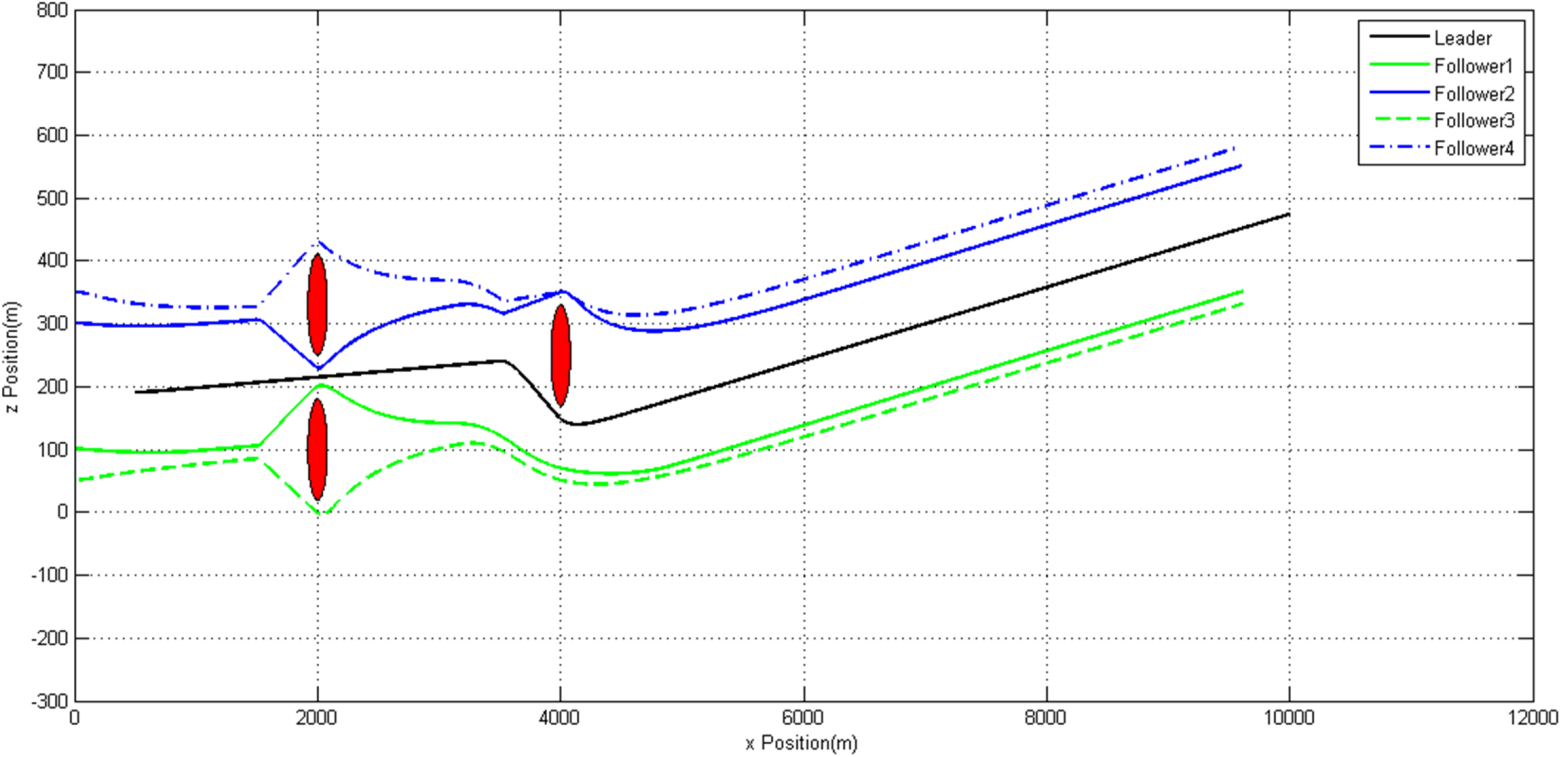
Trajectories of UAVs in *xoz* plane.

Figs 7–9 show the trajectories of each UAV. It can be observed that all the static obstacles are successfully avoided. Though sometimes there is no static obstacle around an UAV, this UAV will manoeuver in order to avoid other UAVs which getting close to it. These prove our modified algorithm can avoid static and dynamic obstacles effectively.

**Fig 10:**
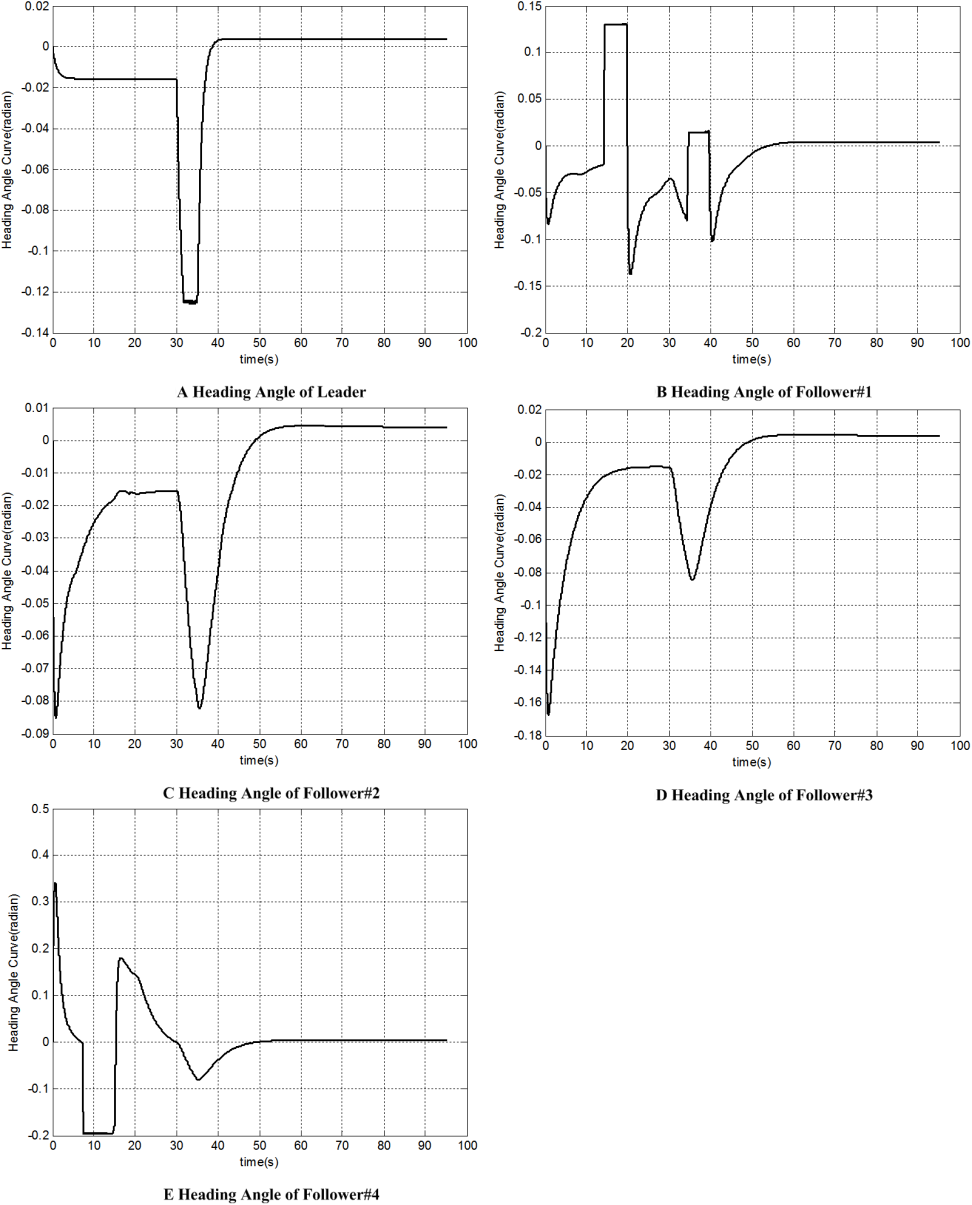
Heading angle histories of each UAV.

**Fig 11:**
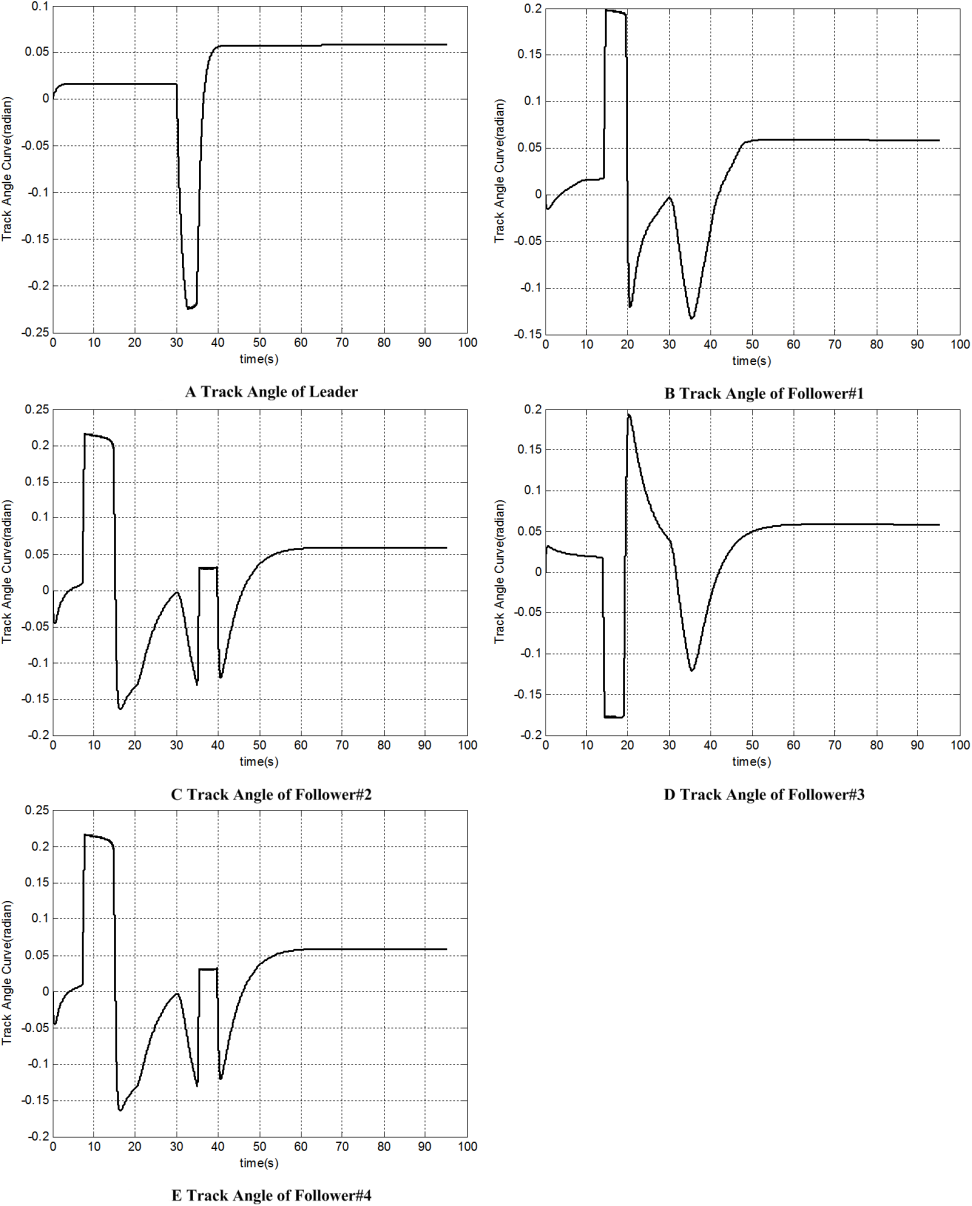
Track angle histories of each UAV.

**Fig 12:**
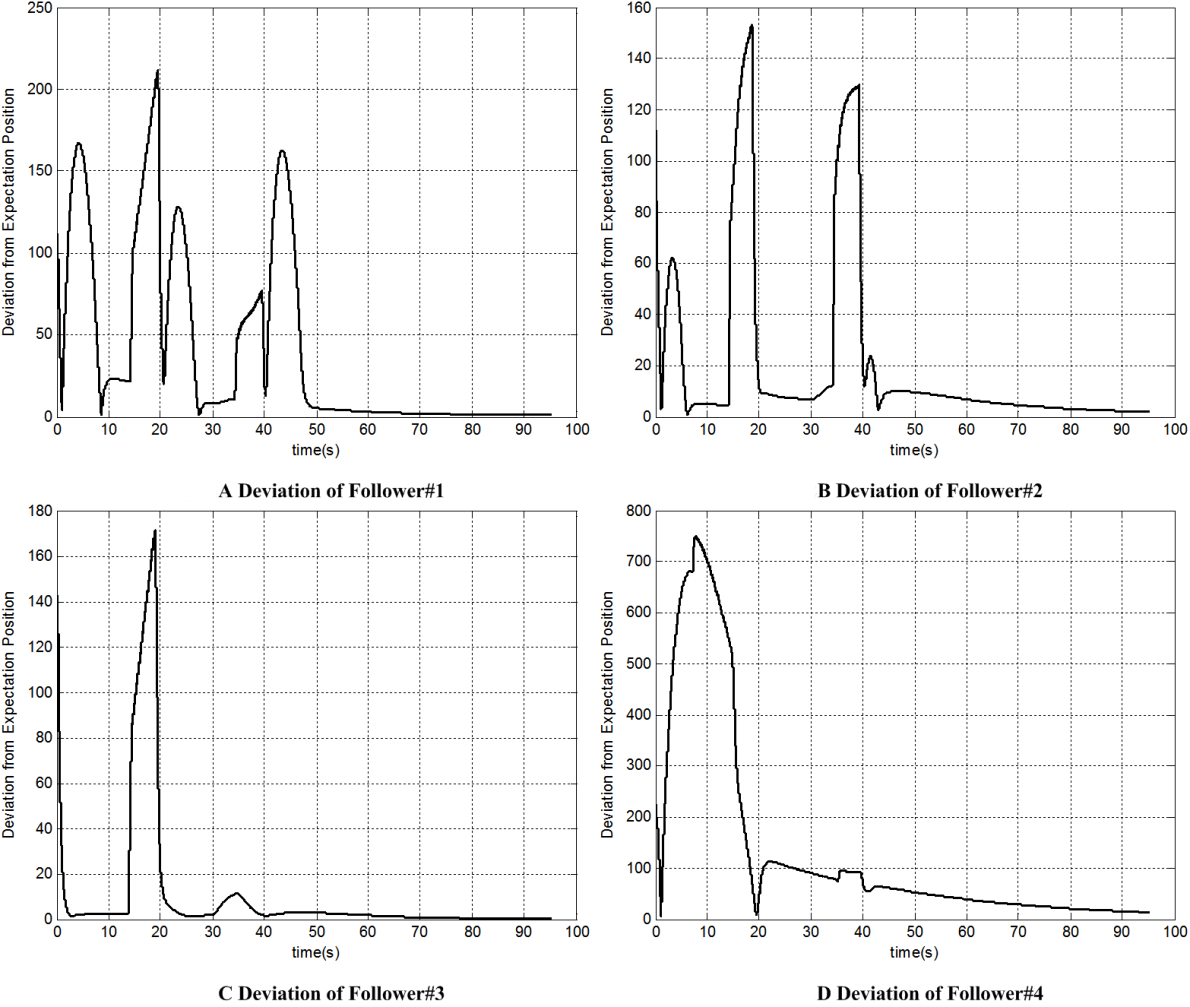
Deviation from expectation position of each UAV.

Figs 10 and 11 give the heading angle and track angle of each UAV; Fig 12 shows the distance from each follower to its formation position. Both of them show that each follower can recover its original formation position after the avoidance.

Meanwhile, each wave crest or wave trough in Figs 10 and 11 shows a large-radius manoeuver. The successful multiple large-radius manoeuver of 5 UAVs prove our inverse derivation solution to the data calculation problem(Problem 1) is credible.

Tables 4 and 5 show the minimum distances among all UAVs and the minimum distances from each UAV to obstacles. The results show that all UAVs can maintain their relative distances farther than the safe distances from other followers and obstacles. These prove that the collision avoidance method proposed in this paper has a high performance.

**Table 4:** The minimum distances among all UAVs.

Parameters	Leader	Follower#1	Follower#2	Follower#3	Follower#4
Leader	0.0m	297.6m	364.4m	512.0m	348.8m
Follower#1	297.6m	0.0m	357.5m	111.4m	505.8m
Follower#2	364.4m	357.5m	0.0m	500.0m	153.5m
Follower#3	512.0m	111.4m	500.0m	0.0m	650.3m
Follower#4	348.8m	505.8m	153.5m	650.3m	0.0m

**Table 5:** The minimum distances from each UAV to obstacles.

Parameters	Leader	Follower#1	Follower#2	Follower#3	Follower#4
Obstacle#1	87.4m	59.7m	221.0m	123.9m	499.1m
Obstacle#2	221.0m	435.5m	82.7m	584.1m	58.7m
Obstacle#3	59.5m	125.5m	180.9m	208.2m	325.5m

Meanwhile, the high-performance collision avoidance method proves that our reduction and reconstruction solution to the application problem (Problem 2) can be solved, and the modified tentacle algorithm can be successfully applied into the collision avoidance of multiple high-speed UAVs.

With the modified tentacle algorithm and other classical collision avoidance methods, Table 6 compares the computational load of path planning and environment modelling in 5 continuous time sets. The comparison results show that the computational load of our algorithm is much less than others, reducing about 50%, thereby proving that our algorithm can effectively compute in real time.

**Table 6:** The computation load in 5 continuous time sets.

Parameters	Set1	Set2	Set3	Set4	Set5
Our algorithm	0.023755s	0.023211s	0.021982s	0.021497s	0.023328s
Artificial potential field method	0.046742s	0.046635s	0.042276s	0.042587s	0.044569s
Grid method	0.050287s	0.049872s	0.049247s	0.048973s	0.051846s

The modified tentacle algorithm are validated in two other scenarios, and the corresponding simulation results are shown in S1 File.

## 5. Conclusion

This paper proposes the modified tentacle algorithm for the formation flight and collision avoidance of multiple UAVs. It concludes that the two problems for applying the conventional tentacle algorithm to the high-speed UAV in unstructured environments: (1) the data calculation problem and (2) the application problem. To solve Problem 1, it modified the tentacle algorithm to rapidly match the radius of each tentacle and the steering command by using the inverse derivation. To solve Problem2, it reduced and reconstructed the speed sets and tentacles in one speed set. By converting global path optimization into local searching, the best tentacle is selected to quickly obtain the UAV collision avoidance path. Consequently, the overall formation flight and collision avoidance mission are guaranteed simultaneously, so that the high-speed UAV formation can avoid collision in unstructured environments through applying our collision avoidance method. The simulation results in two scenarios do indeed confirm the feasibility and effectiveness of the method. The results also show that the multiple UAVs can cope with other collision threats coming from other UAVs in the formation and unknown obstacles. Multiple-UAV formation flight and collision avoidance can be achieved by data calculation at every moment. Therefore, the collision avoidance method can effectively compute in real time the collision avoidance of multiple high-speed UAVs under unstructured environments.

## Supporting information

S1 File Supporting information. (PDF)
