# Supplementary material for: Formation flight and collision avoidance for multiple UAVs based on modified tentacle algorithm in unstructured environments
Source: PLoS One. 2017 Aug 1;12(8):e0182006. doi: 10.1371/journal.pone.0182006 (PMC5538717; doi:10.1371/journal.pone.0182006)
Supplement: S1 File — (PDF) [file pone.0182006.s001.pdf]

Actually, many scenarios are created to verify my algorithm in the simulation part. Because there are too many figures and tables, I just show one typical scenario in my paper. Here I will show the multi-UAVs collision avoidance in other two scenarios.

Scenario 1

The leader-follower formation model contains one leader and four followers. The initial states of each UAV are given in Table 1, where $\tau_v, \tau_\psi, \tau_\theta$ represent the time constants of the autopilot.

Table 1 UAV's initial states

Parameters	Position(m)	Speed (m/s)	Heading angle(Degree)	Track angle(Degree)	τ_v	τ_ψ	τ_θ
Leader	(500,200,190)	100	0	0	5	1	1
Follower#1	(0,0,100)	100	0	0	5	1	1
Follower#2	(0,300,300)	100	0	0	5	1	1
Follower#3	(0,-200,50)	100	0	0	5	1	1
Follower#4	(0,700,350)	100	0	0	5	1	1

Table 2 shows the relative distance in three dimensions between each follower and leader:

Table 2 Formation flight requirements

Parameters	Relative Distance in the X Direction (m)	Relative Distance in the Y Direction (m)	Relative Distance in the Z Direction (m)
Leader	0	0	0
Follower#1	400	150	100
Follower#2	400	-150	-100
Follower#3	400	300	120
Follower#4	400	-300	-130

Table 3 gives the information on obstacles during the simulation, where d_s represents the requirements for safe distances in each tentacle.

Table 3 Obstacle parameters

Parameters	Position(m)	Radius(m)	Safe distance ds in tentacle(m)
Obstacle#1	(4000,120,100)	80	30
Obstacle#2	(4000,500,330)	80	30
Obstacle#3	(2000,200,250)	80	30

The simulation will be terminated when the leader UAV reach 10000m in x direction. The simulation step is 0.001s. Simulation results are as follows:

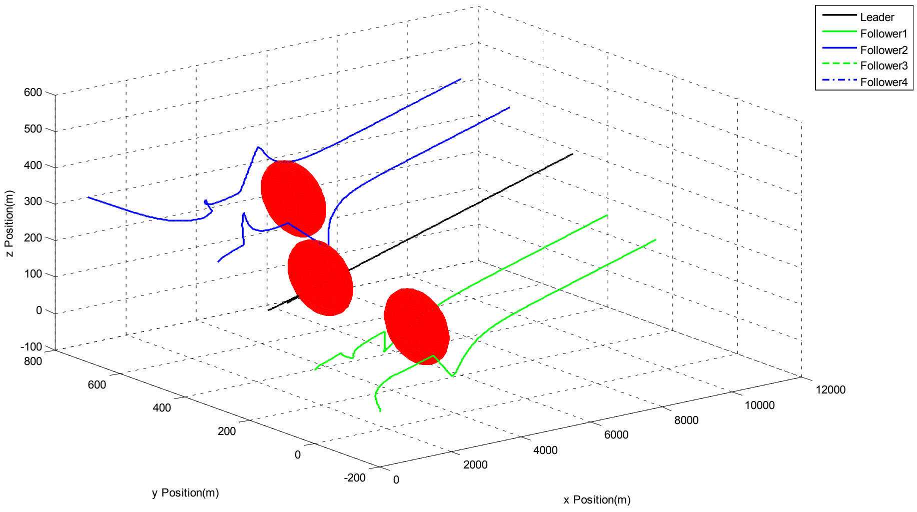

Fig 1 3D Trajectories of UAVs

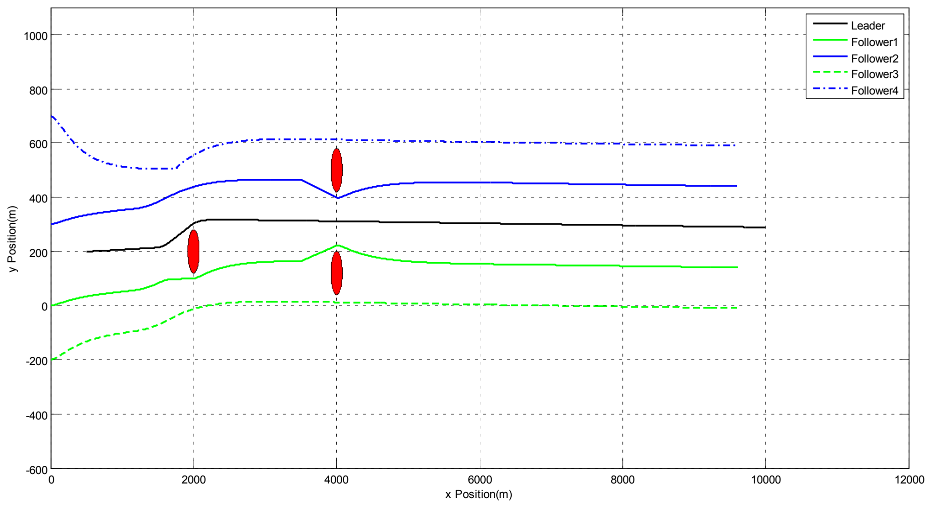

Fig 2 Trajectories of UAVs in *xoy* plane

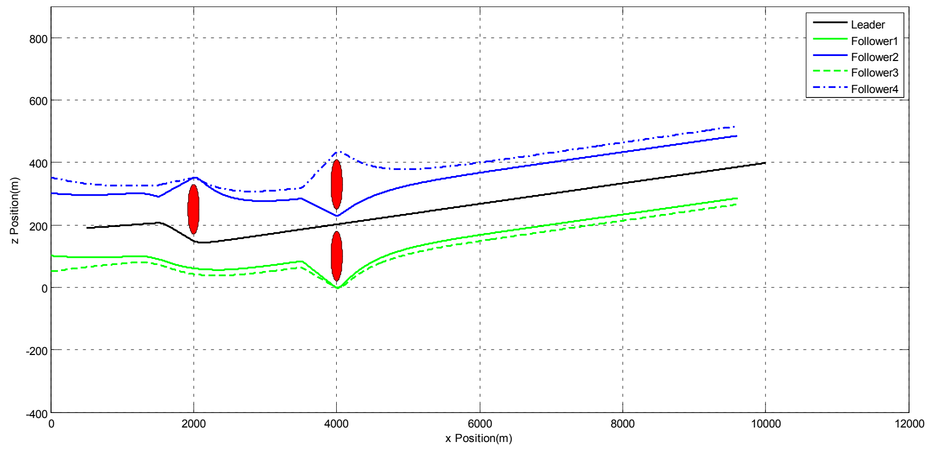

Fig 3 Trajectories of UAVs in *xoz* plane

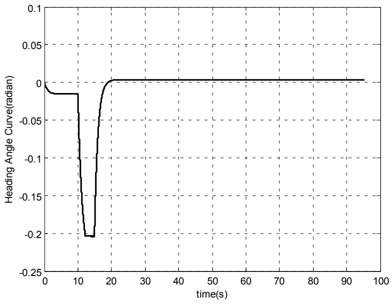

Fig 4A Heading Angle of Leader

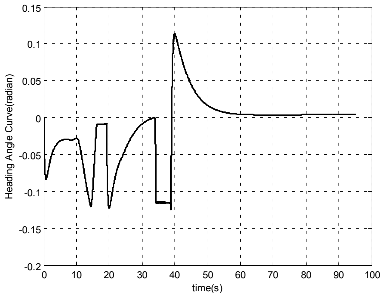

Fig 4B Heading Angle of Follower#1

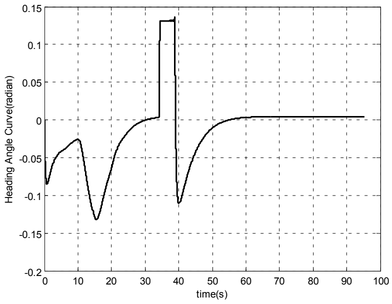

Fig 4C Heading Angle of Follower#2

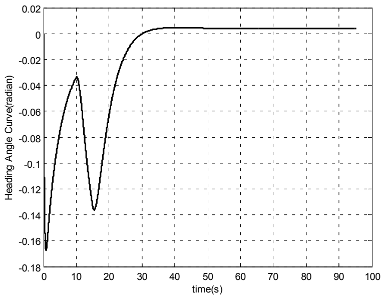

Fig 4D Heading Angle of Follower#3

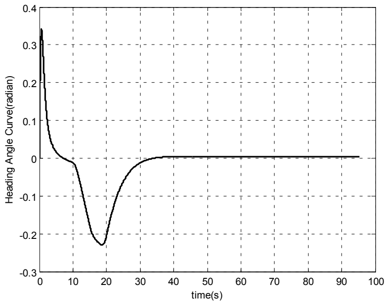

Fig 4E Heading Angle of Follower#4

Fig 4 Heading Angle Histories of each UAV

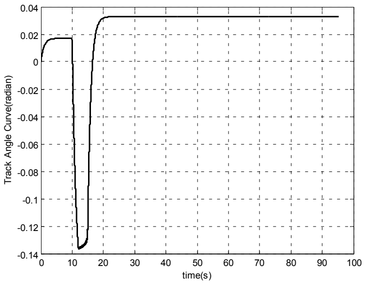

Fig 5A Track Angle of Leader

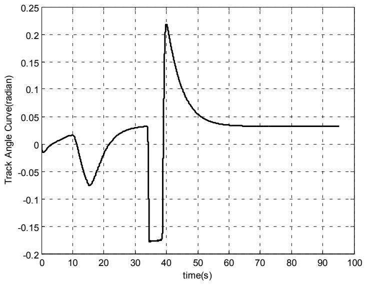

Fig 5B Track Angle of Follower#1

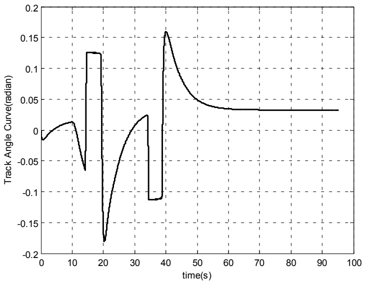

Fig 5C Track Angle of Follower#2

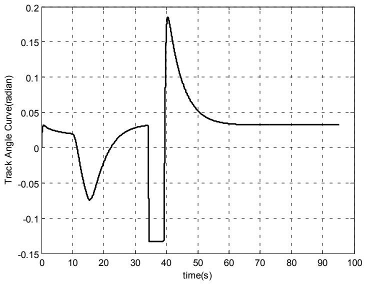

Fig 5D Track Angle of Follower#3

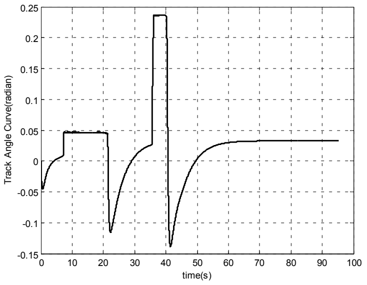

Fig 5E Track Angle of Follower#4

Fig 5 Track Angle Histories of each UAV

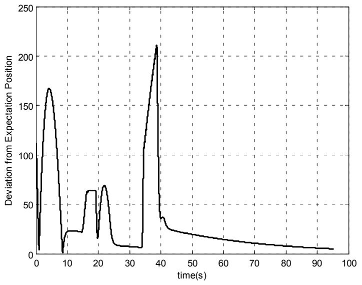

Fig 6A Deviation of Follower#1

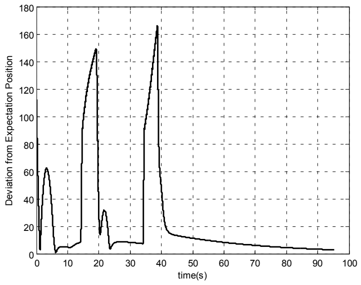

Fig 6B Deviation of Follower#2

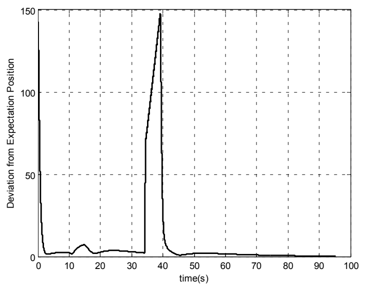

Fig 6C Deviation of Follower#3

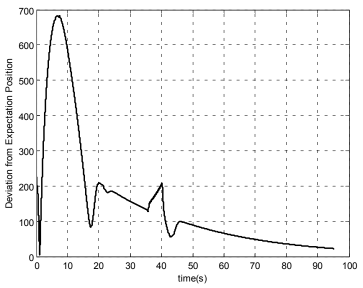

Fig 6D Deviation of Follower#4

Fig 6 Deviation from Expectation Position of each UAV

Table 4 The Minimum Distances among all UAVs

Parameters	Leader	Follower#1	Follower#2	Follower#3	Follower#4
Leader	0.0m	297.6m	379.5m	511.9m	299.0m
Follower#1	297.6m	0.0m	290.5m	113.5m	491.5m
Follower#2	379.5m	290.5m	0.0m	449.1m	94.9m
Follower#3	511.9m	113.5m	449.1m	0.0m	614.7m
Follower#4	299.0m	491.5m	94.9m	614.7m	0.0m

Table 5 The Minimum Distances from each UAV to Obstacles

Parameters	Leader	Follower#1	Follower#2	Follower#3	Follower#4
Obstacle#1	136.5m	60.0m	226.9m	66.9m	509.6m
Obstacle#2	148.3m	352.8m	60.4m	507.6m	70.6m
Obstacle#3	59.6m	133.7m	176.1m	217.0m	282.6m

Scenario 2

The leader-follower formation model contains one leader and four followers. The initial states of each UAV are given in Table 6, where $\tau_v, \tau_\psi, \tau_\theta$ represent the time constants of the autopilot.

Table 6UAV's initial states

Parameters	Position(m)	Speed (m/s)	Heading angle(Degree)	Track angle(Degree)	τ_v	τ_ψ	τ_θ
Leader	(500,200,190)	100	0	0	5	1	1
Follower#1	(0,0,100)	100	0	0	5	1	1
Follower#2	(0,300,300)	100	0	0	5	1	1
Follower#3	(0,-200,50)	100	0	0	5	1	1
Follower#4	(0,700,350)	100	0	0	5	1	1

Table 7 shows the relative distance in three dimensions between each follower and leader:

Table 7 Formation flight requirements

Parameters	Relative Distance in the X Direction (m)	Relative Distance in the Y Direction (m)	Relative Distance in the Z Direction (m)
Leader	0	0	0
Follower#1	400	150	100
Follower#2	400	-150	-100
Follower#3	400	300	120
Follower#4	400	-300	-130

Table 8 gives the information on obstacles during the simulation, where d_s represents the requirements for safe distances in each tentacle.

Table 8 Obstacle parameters

Parameters	Position(m)	Radius(m)	Safe distance ds in tentacle(m)
Obstacle#1	(4000,100,100)	80	30
Obstacle#2	(3000,300,230)	80	30
Obstacle#3	(2000,200,250)	80	30

The simulation will be terminated when the leader UAV reach 10000m in x direction. The simulation step is 0.001s. Simulation results are as follows:

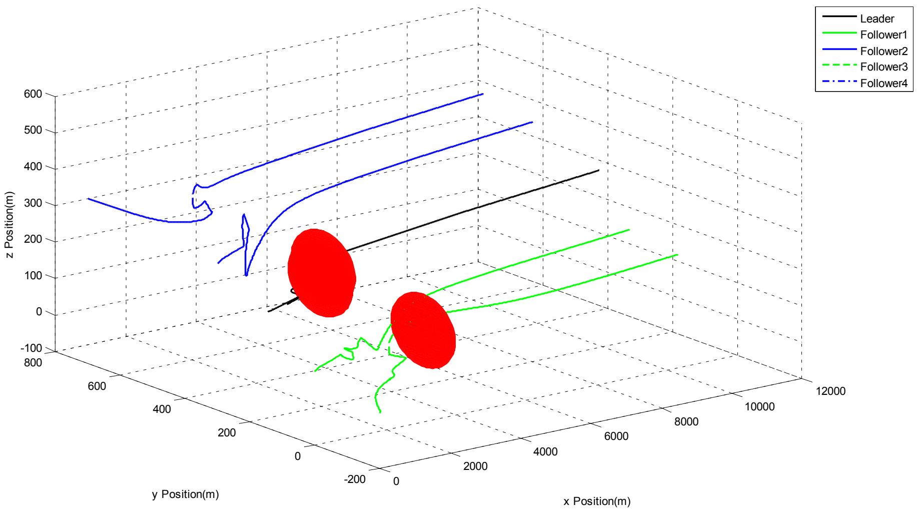

Fig 7 3D Trajectories of UAVs

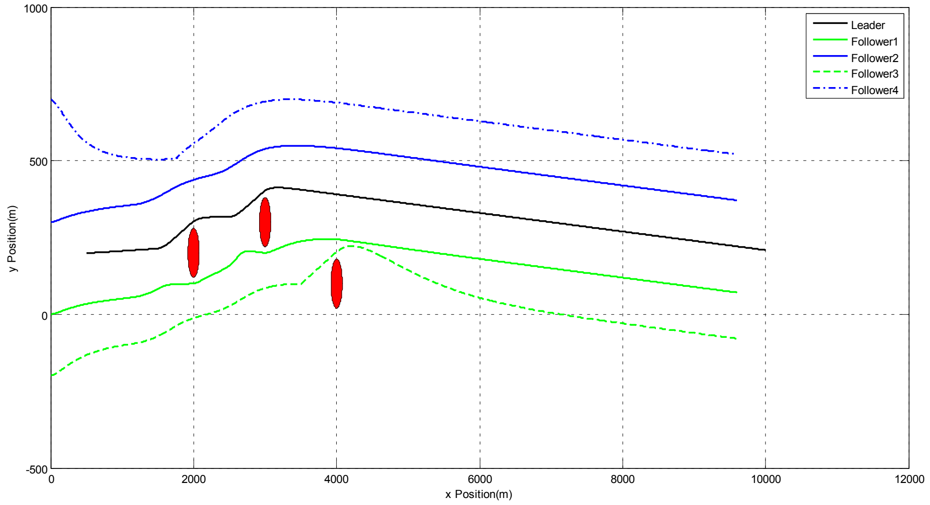

Fig 8 Trajectories of UAVs in xoy plane

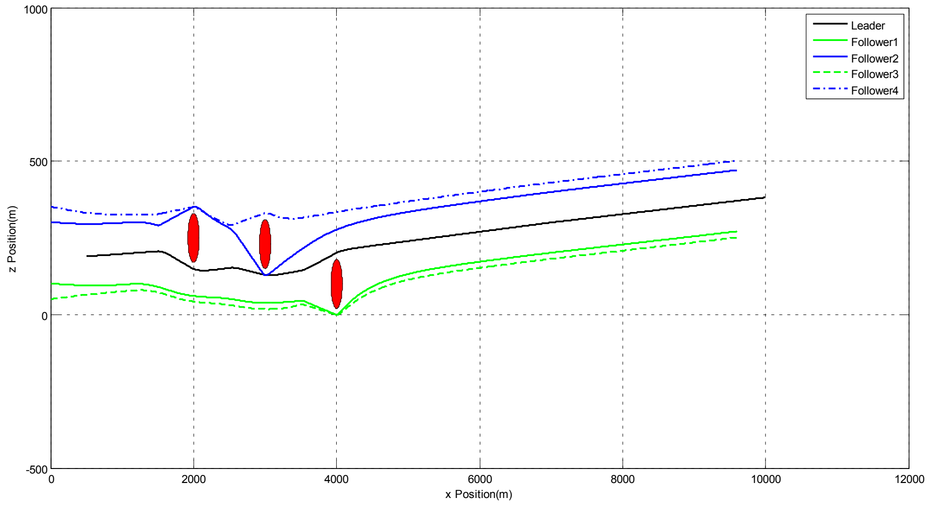

Fig 9 Trajectories of UAVs in *xoz* plane

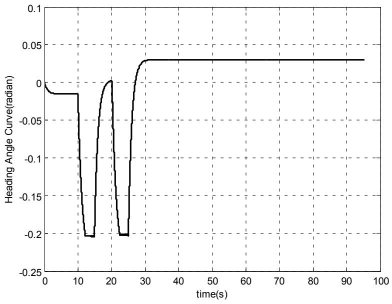

Fig 10A Heading Angle of Leader

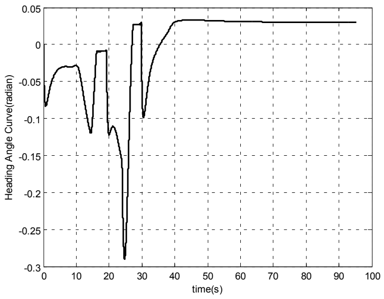

Fig 10B Heading Angle of Follower#1

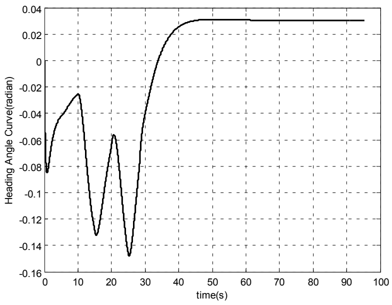

Fig 10C Heading Angle of Follower#2

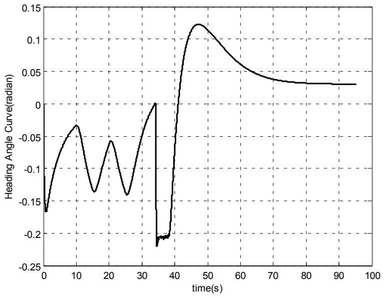

Fig 10D Heading Angle of Follower#3

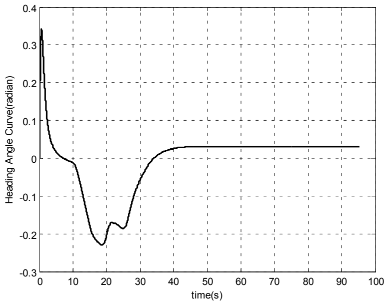

Fig 10E Heading Angle of Follower#4

Fig 10 Heading Angle Histories of each UAV

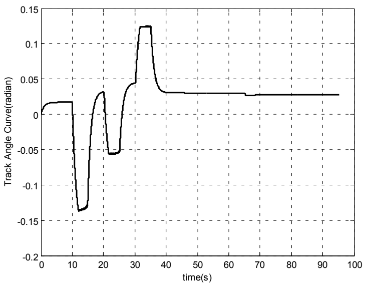

Fig 11A Track Angle of Leader

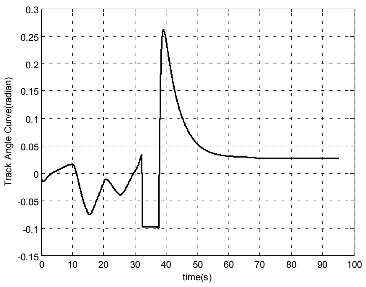

Fig 11B Track Angle of Follower#1

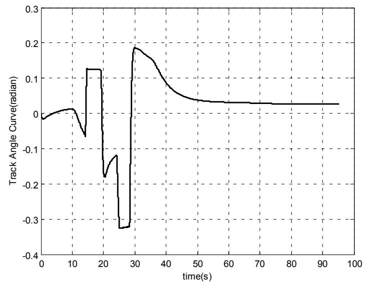

Fig 11C Track Angle of Follower#2

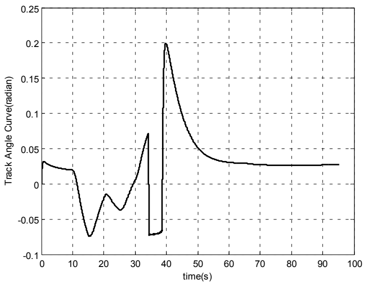

Fig 11D Track Angle of Follower#3

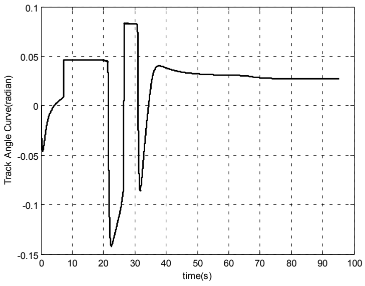

Fig 11E Track Angle of Follower#4

Fig 11 Track Angle Histories of each UAV

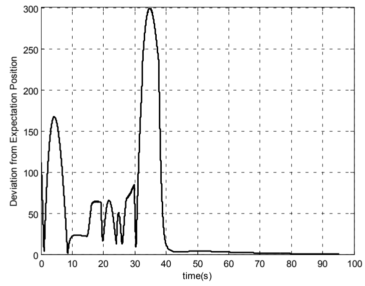

Fig 12A Deviation of Follower#1

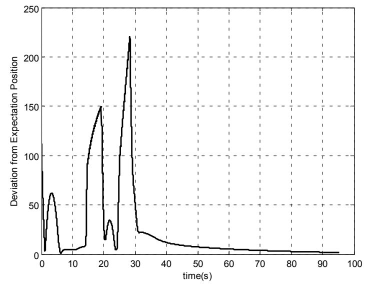

Fig 12B Deviation of Follower#2

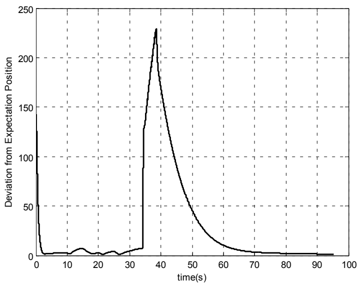

Fig 12C Deviation of Follower#3

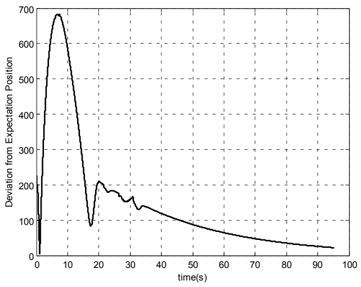

Fig 12D Deviation of Follower#4

Fig12 Deviation from Expectation Position of each UAV

Table 9 The Minimum Distances among all UAVs

Parameters	Leader	Follower#1	Follower#2	Follower#3	Follower#4
Leader	0.0m	254.3m	363.6m	419.9m	299.0m
Follower#1	254.3m	0.0m	350.7	19.3m	491.5m
Follower#2	363.6m	350.7m	0.0m	399.3m	94.9m
Follower#3	419.9m	19.3m	399.3m	0.0m	554.7m
Follower#4	299.0m	491.5m	94.9m	554.7m	0.0m

Table 10 The Minimum Distances from each UAV to Obstacles

Parameters	Leader	Follower#1	Follower#2	Follower#3	Follower#4
Obstacle#1	227.8m	95.6m	394.7m	60.5m	554.0m
Obstacle#2	60.7m	135.9m	175.5m	221.7m	324.3m
Obstacle#3	59.6m	133.7m	176.1m	217.0m	282.8m
